# Recent trends and perspectives of molecular markers against fungal diseases in wheat

**DOI:** 10.3389/fmicb.2015.00861

**Published:** 2015-08-25

**Authors:** Umesh Goutam, Sarvjeet Kukreja, Rakesh Yadav, Neha Salaria, Kajal Thakur, Aakash K. Goyal

**Affiliations:** ^1^Department of Biotechnology, Lovely Professional University, PhagwaraPunjab, India; ^2^Department of Bio and Nano technology, Guru Jambheshwar University of Science and TechnologyHisar, India; ^3^International Center for Agriculture Research in the Dry Areas (ICARDA)Morocco

**Keywords:** MAS, molecular markers, R genes, wheat, wheat rust

## Abstract

Wheat accounts for 19% of the total production of major cereal crops in the world. In view of ever increasing population and demand for global food production, there is an imperative need of 40–60% increase in wheat production to meet the requirement of developing world in coming 40 years. However, both biotic and abiotic stresses are major hurdles for attaining the goal. Among the most important diseases in wheat, fungal diseases pose serious threat for widening the gap between actual and attainable yield. Fungal disease management, mainly, depends on the pathogen detection, genetic and pathological variability in population, development of resistant cultivars and deployment of effective resistant genes in different epidemiological regions. Wheat protection and breeding of resistant cultivars using conventional methods are time-consuming, intricate and slow processes. Molecular markers offer an excellent alternative in development of improved disease resistant cultivars that would lead to increase in crop yield. They are employed for tagging the important disease resistance genes and provide valuable assistance in increasing selection efficiency for valuable traits via marker assisted selection (MAS). Plant breeding strategies with known molecular markers for resistance and functional genomics enable a breeder for developing resistant cultivars of wheat against different fungal diseases.

## Introduction

Wheat is a major staple food for mankind in many parts of the world with 714 million tons produced during 2013 (http://www.agri-outlook.org). It is cultivated on 15.4% of the arable land in the world in almost all countries, except the humid and high-temperature areas in the tropics and high-latitude environments. Accounting for a fifth of humanity’s food, wheat is the second only to rice which provides 21% of the food calories and 20% of the protein for more than 4.5 billion people in 94 developing countries (Braun et al., 2010). It contributes 30% of the world’s edible dry matter and 60% of the daily calorie intake in several developing countries (FAOSTAT, 2015). Wheat is produced for a wide range of end-users and it is a critical staple food for a large proportion of the world’s poor farmers and consumers. Due to consistent increase in the world population, there is a need of 60% increase in wheat production to meet the requirement of developing world till 2050 (Singh and Trethowan, 2007; Singh et al., 2007; Rosegrant and Agcaoili, 2010).

Increasing wheat yield potential in the developing world is a primary aim for food security concern (Duveiller et al., 2007). Today, the most challenging task for wheat breeders is to increase grain yield as well as to improve the grain quality of crop for end products (Goutam et al., 2013). These two aspects must be cope up with the strategies employed for enhancing the tolerance against biotic (Keller et al., 2008; Todorovska et al., 2009) and abiotic stresses (Kamal et al., 2010) in addition to the enhanced capability to adapt to various climate changes (Olmstead and Rhode, 2011). Amongst the most important diseases in wheat (derived from fungi, virus, and bacteria), rust diseases (leaf, stem, and stripe) caused by fungus, powdery mildew and Karnal bunt have been reported to produce devastating consequences on wheat quality and production (Keller et al., 2008; Goyal and Prasad, 2010). Cereal rust fungi are highly variable for virulence and molecular polymorphism. Leaf rust, caused by *Puccinia triticina* is the most common rust of wheat on a worldwide basis (Kolmer, 2013). Leaf rust has potential to cause losses of up to 50% and because of its more frequent and widespread occurrence, leaf rust probably results in greater total annual losses worldwide than stem and stripe rusts (Huerta-Espino et al., 2011). However, management of fungal diseases using conventional plant protection and breeding strategies is quite easy and effective tool, but, it results into different types of environmental pollutions as it involves the use of various eco-hazardous chemicals. Identification and selection of resistant genes through breeding practices is also time-consuming and slow process. Moreover, disease management by host resistance, employment of stable diseases resistance and development of homozygous and resistant cultivars are also time consuming methods (Sharma, 2003; Keller et al., 2008).

To overcome these problems, molecular marker technology is the novel genetic tool for developing high yielding disease resistant cultivars (Landjeva et al., 2007; Varshney et al., 2007). Molecular markers could tag the presence of important resistance genes and allow breeders to identify the resistance genes rapidly and accurately. They also provide significant assistance for increasing selection efficiency through indirect selection for valuable traits via marker assisted selection (MAS). Thus, MAS offers a potential tool for assisting conventional plant breeding approaches to select phenotypic traits for screening disease resistant crop plants (Todorovska et al., 2009). Therefore, existing plant breeding techniques along with available molecular markers (Gupta et al., 2010) and functional genomic tools (Gupta et al., 2008) can help a breeder for developing superior wheat cultivars resistant against fungal diseases in order to minimize yield losses (Goyal and Prasad, 2010). Different types of markers such as random DNA markers, gene targeted markers (Gupta et al., 2010) and functional markers (Liu et al., 2012) have been reported for facilitating identification of genes responsible for individual traits and for improving potential of using MAS in wheat breeding programs (Gupta et al., 2008). DNA-based molecular markers like RFLP (Hartl et al., 1993; Ma et al., 1993, 1994; Autrique et al., 1995; Paull et al., 1995; Nelson et al., 1997), RAPD (Penner et al., 1995; Procunier et al., 1995; Demeke et al., 1996; Qi et al., 1996; Dweikat et al., 1997; Dubcovsky et al., 1998; Shi et al., 1998), STS (Schachermayr et al., 1994, 1995, 1997; Feuillet et al., 1995; Dedryver et al., 1996; Naik et al., 1998; Prins et al., 2001), SSR (Peng et al., 2000; Raupp et al., 2001; Wang et al., 2002), CAPS (Helguera et al., 2000, 2003), AFLP (Hartl et al., 1998), and SCAR (Gold et al., 1999; Liu et al., 1999) have been commonly used for the molecular characterization of plant pathogen and mapping of disease resistance genes in wheat. The development of plant gene transfer systems enable us for the introgression of foreign genes into plant genomes for novel disease control strategies, thus providing a mechanism for broadening the genetic resources available to plant breeders (Zhu et al., 2012).

## Fungal Diseases of Wheat

Worldwide, wheat diseases caused by fungal pathogens are more threatening for crop yields and grain quality than those caused by bacteria and viruses. Since, the fungal pathogens are very adaptable and can rapidly evolve into new strains that can infect earlier disease resistant plants. Infection of wheat fungal diseases are influenced by various factors viz., nature of pathogen, susceptibility of host, diversity of virulence, density of inoculums and temperature (Rajaram and Van Ginkel, 1996; McIntosh et al., 1998). The most important fungal diseases in wheat include different types of rust, powdery mildew and Karnal bunt.

**Key concepts**

**(1) DNA marker**

It is a gene or DNA sequence with a known location on a chromosome that can be used to identify individuals or species. A genetic marker may be a short DNA sequence, such as a sequence surrounding a single base-pair change (single nucleotide polymorphism, SNP), or a long one, like minisatellites.

**(2) Fungal disease**

An abnormal growth and/or dysfunction of a plant caused by fungi, which disturbs the normal life process of the plant.

**(3) Marker assisted selection (MAS)**

MAS is a process whereby a marker (morphological, biochemical or one based on DNA/RNA variation) is used for indirect selection of a genetic determinant or determinants of a trait of interest (e.g., productivity, disease resistance, abiotic stress tolerance, and quality).

**(4) Wheat rust**

Wheat rust is a destructive disease of wheat caused by fungus genus *Puccinia*, especially a destructive stem rust characterized by reddish blisters that turn black at the end of the growing season.

## Wheat Rust

Wheat rust pathogens belong to genus *Puccinia*, family *Pucciniaceae*, order Uredinales and class Basidiomycetes. The rust diseases of wheat such as leaf rust, stem rust, and stripe rust have historically been among the major biotic constraints in the world (Saari and Prescott, 1985; Todorovska et al., 2009). The rusts of wheat is caused by fungal pathogens that can be disseminated thousands of kilometers by wind and are capable of causing considerable economic loss throughout the world (Kolmer, 2005; Goyal and Prasad, 2010). The importance of genetic resistance for the control of rust diseases was demonstrated by Biffen (1905). A prerequisite for developing cultivars with long term rust resistance is the availability of diverse resistance genes.

## Leaf Rust

Leaf rust, also known as brown rust, is caused by fungus *P. triticina* Rob. Ex Desm. f. sp. *tritici* Eriks (syn. *P. recondita*). It is a wheat disease of major historical and economic importance. Leaf rust is the most prevalent amongst all the wheat rust diseases occurring around nearly in all wheat grown areas (Kolmer, 2005; Huerta-Espino et al., 2011; Vanzetti et al., 2011). Therefore, it is considered as a widespread and commonly occurring rust disease of wheat. The disease has caused serious epidemics in wheat growing regions of USA (Appel et al., 2009), North Western Mexico (Dubin and Torres, 1981; Singh, 1991; Singh et al., 2004), South America (German et al., 2004), Northern Africa (Abdel-Hak et al., 1980; Deghais et al., 1999), Russia (Volkova et al., 2009), India (Joshi et al., 1975; Nagarajan and Joshi, 1978), Pakistan (Hassan et al., 1973; Hussain et al., 1980), Australia (Watson and Luig, 1961; Keed and White, 1971; Rees and Platz, 1975; Murray and Brennan, 2009), South Africa (Terefe et al., 2009) and other parts of the world. Leaf rust is generally localized on the leaves, but occasionally affects the glumes and awns. Symptoms include circular or oval, orange pustules (urediniospores) on the upper surface of infected leaves. Later on, these pustules become darker due to the formation of black telliospores (Roberson and Luttrell, 1987). The loss in yield depends on several factors such as time of initial infection, crop development stages and relative resistance or susceptibility of the wheat cultivars. Higher yield losses materialized if the initial infection occurs early in the growing season before tillering. However, infection occurred after heading when grain filling is in progress, will cause lesser crop loss (Agrios, 1997). Wheat yield losses are caused due reduction in number of kernels per spike, and kernel weight. Depending on the severity and duration of infection, the losses can vary up to 50% in susceptible wheat cultivars (Knott, 1989; McIntosh et al., 1995).

More than 60 leaf rust-resistance (*Lr*) genes have been identified in common wheat, durum wheat and diploid wheat species (McIntosh et al., 1995, 2008; Bansal et al., 2008; Chhuneja et al., 2008; Vida et al., 2009). Majority of the genes have been identified in the wild wheat relative *Aegilops tauschii* (Rowland and Kerber, 1974; Kerber, 1987; Gill et al., 1991; Cox et al., 1994; Huang and Gill, 2001; Raupp et al., 2001; Huang et al., 2003; Hiebert et al., 2007). Breeding for leaf rust resistance in wheat is the most challenging task for a breeder because resistance can be completely defeated by a shift in predominant pathogen race in a rust population. Therefore, use of genetic resistance is the comparatively promising option to combat rust epidemics in crop plants. Genetic resistance has two dimensions; one is monitoring dynamic changes of rust pathogen populations to identify new virulent races, and second is deploying resistance genes to defeat the new pathogen race. Molecular markers viz., RFLP, RAPD, STS, SCAR, CAPS, and SSR proves to be the best alternative for screening against leaf rust resistance (William et al., 2008). A wide range of markers are reported to be associated with *Lr* genes (**Table 1**). RFLP (*Lr13*-Seyfarth et al., 2000; *Lr20*-Neu et al., 2002; *Lr21*-Huang and Gill, 2001; *Lr23, Lr27*-Nelson et al., 1997; *Lr24, Lr32*-Autrique et al., 1995) and RAPD (*Lr25, Lr29*-Procunier et al., 1995) have been used to tag a variety of *Lr* genes in wheat. Moreover, the conversion of RFLPs and RAPDs into STS (Schachermayr et al., 1994, 1995, 1997; Feuillet et al., 1995; Helguera et al., 2005) or SCARs (Dedryver et al., 1996) provided a range of useful markers for *Lr* genes. STS or SCARs are the preferred DNA markers over RFLP, RAPD and AFLP. *Lr1* (Feuillet et al., 1995), *Lr9, Lr10* (Schachermayr et al., 1994, 1995, 1997), *Lr19* (Prins et al., 2001; Cherukuri et al., 2003), *Lr24* (Schachermayr et al., 1995; Dedryver et al., 1996), *Lr28* (Naik et al., 1998), *Lr35* (Gold et al., 1999; Seyfarth et al., 1999), *LrX* (Obert et al., 2005), *Lr51* (Helguera et al., 2005) and *Lr* 26 (Zhou et al., 2014) are the different STS or SCAR markers associated to *Lr* genes. *Lr67* (Hiebert et al., 2010) and *Lr68* (Herrera-Foessel et al., 2012) are SSR linked *Lr* genes. A gene *TaHIR3* has been characterized which encodes a hypersensitive-induced reaction (HIR) protein in response to pathogen attacks. Its expression profile at the DNA and protein levels suggested that *TaHIR3* and its deduced protein play a significant role in wheat hypersensitive response caused by leaf rust pathogen (Yu et al., 2013). Validation of markers linked to resistance genes was done successfully in wheat germplasm worldwide. The 287 BC_2_F_4_ population of Hungarian wheat genotypes ‘Mv Emma’^∗^3/‘R.L.6010’ was tested for the presence of *Lr (Lr9, Lr24, Lr 25, Lr 29, Lr35, and Lr37)* genes. SCAR markers were used for screening of *Lr24, Lr 25*, and *Lr 37* genes (Robert et al., 1999), whereas, STS and RAPD markers were used to validate the presence of *Lr 9, Lr 35*, and *Lr 29*, respectively (Vida et al., 2009). Prabhu et al. (2003) used RAPD and SSR marker to study presence of *Lr 32* and *Lr 28*, respectively, in 10 elite near-isogenic lines (NILs) of Indian bread wheat genotypes. To identify the resistance genes in 23 hexaploid Russian spring wheat, STS markers linked to the known leaf rust resistance genes *Lr1, Lr9, Lr10, Lr21, Lr24, Lr28, Lr35, Lr37*, and *Lr39* were used (Gajnullin et al., 2007). Gene-specific markers to the seedling resistance genes (*Lr1, Lr10*, and *Lr21*) and Adult plant resistance gene (*Lr34*) were utilized for molecular screening of 275 wheat accessions from 42 countries (Dakouri et al., 2013). Imbaby et al. (2014) conducted study to identify *Lr13, Lr19, Lr24, Lr26, Lr34, Lr35, Lr36, Lr37, Lr39*, and *Lr46* in 15 Egyptian wheat cultivars using various types of molecular markers.

**Table 1:** List of Molecular markers linked to major fungal disease resistance genes.

Trait	Locus	Marker	Source	Donor	Reference
Leaf rust	*Lr1*	RFLP/STS	*Triticum aestivum*	ThatcherLr1	Feuillet et al. (1995), Qiu et al. (2007)
	*Lr3*	RFLP	*T. aestivum*	Sinvalocho MA	Sacco et al. (1998)
	*Lr3a*		*T. aestivum*	Schomburgk and Yarralinka	Khan et al. (2005)
	*Lr9*	RAPD/STS, RFLP	*Aegilops umbellulata*	RL6010	Schachermayr et al. (1994), Autrique et al. (1995), Gupta et al. (2005)
	*Lr10*	RFLP/STS, STS	*T. aestivum*	Thatcher Lr10	Schachermayr et al. (1997), Feuillet et al. (2003), Stepien et al. (2003)
	*Lr12*	SSR	*T. aestivum*	TcLr12	Singh and Bowden (2011)
	*Lr13*	RFLP, SSR	*T. aestivum*	Thatcher^∗^Frontana	Seyfarth et al. (1998, 1999), Bansal et al. (2008)
	*Lr14*	SSR	*T. aestivum*		Herrera-Foessel et al. (2007)
	*Lr14a*	SNP	*T. durum*	Colosseo	Terracciano et al. (2013)
	*Lr15*	SSR	*T. aestivum*	Tc-Lr15	Dholakia et al. (2013)
	*Lr16*	SSR	*T. aestivum*	BW278	Mccartney et al. (2005)
	*Lr19*	STS, RAPD/SSR	*Agropyron Elongatum*		Prins et al. (2001), Gupta et al. (2006)
	*Lr20*	RFLP	*T. aestivum*	Axminster	Neu et al. (2002)
	*Lr21*	RFLP; KASPar	*T. tauschii*		Huang and Gill (2001), Neelam et al. (2013)
	*Lr22a*	SSR	*T. tauschii*	RL5404	Hiebert et al. (2007)
	*Lr23*	RFLP	*T. turgidum*		Nelson et al. (1997)
	*Lr24*	RFLP, RAPD/STS, RAPD/SCAR, SCAR	*Agropyron elongatum*	Agent	Autrique et al. (1995), Schachermayr et al. (1995), Dedryver et al. (1996), Prabhu et al. (2004)
	*Lr25*	RAPD/SSR	*S. cereale*	TcLr25	Procunier et al. (1995), Singh et al. (2012)
	*Lr26*	SCAR, SSR	*Secale cereale*	Pavon	Mago et al. (2002), Zhou et al. (2014)
	*Lr27*	RFLP, SSR	*T. aestivum*		Nelson et al. (1997), Spielmeyer et al. (2003)
	*Lr28*	STS, SCAR	*T. aestivum*	HD2285	Naik et al. (1998), Cherukuri et al. (2003)
	*Lr29*	RAPD	*Agropyron elongatum*		Procunier et al. (1995)
	*Lr31*	RFLP, SSR	*T. aestivum*		Nelson et al. (1997)
	*Lr32*	RFLP	*T. tauschii*	RL57 1 3	Autrique et al. (1995)
	*Lr34*	STS	*T. aestivum*	Parula7D	Lagudah et al. (2006, 2009), Bossolini et al. (2007)
	*Lr35*	SCAR, STS	*A. Speltoides, T. speltoides*	R.L.6082	Gold et al. (1999), Seyfarth et al. (1999)
	*Lr37*	STS/CAPS, ISSR	*A. Ventricosa*		Helguera et al. (2003)
	*Lr38*	SSR	*Thinopyrum intermedium*	RL6097	Mebrate et al. (2008)
	*Lr39*	SSR	*T. Tauschii*	TA4186	Raupp et al. (2001)
	*Lr41*		*T. Tauschii*	Century	Sun et al. (2009)
	*Lr45*	AFLP,SSR	*T. aestivum*	TcLr45, Thatcher	Zhang et al. (2005), Naik et al. (2015)
	*Lr46*	STS	*T. aestivum*	Pavon	Mateos-Hernandez et al. (2006)
	*Lr47*	RFLP, CAPS	*T.speltoides*	Tausch, T7AS-7S#1S-7AS⋅7AL	Dubcovsky et al. (1998), Helguera et al. (2000)
	*Lr48*	SSR	*T. aestivum*	CSP44/WL711 VL404/WL711	Bansal et al. (2008)
	*Lr49*	SSR	*T. aestivum*	CSP44/WL711 VL404/WL711	Bansal et al. (2008)
	*Lr50*	SSR	*T. timopheevii*		Brown-Guedira et al. (2003)
	*Lr51*	STS	*T. speltoides*		Helguera et al. (2005)
	*Lr52*	STS	*T. aestivum*	RL6107	Tar et al. (2008)
	*Lr 58*	SSR	*T. aestivum*	TA5605	Kuraparthy et al. (2007b)
	*Lr60*	SSR	*T. aestivum*		Hiebert et al. (2008)
	*Lr63*	SSR	*T. monococcum*	RL6137	Kolmer (2008)
	*Lr64*	SSR	*T. dicoccoides*		Kolmer (2008)
	*Lr67*	SSR	*T. aestivum*	Thatcher/RL6077 RL6058/RL6077	Hiebert et al. (2010)
	*Lr68*	SSR, CAPS	*T. aestivum*	Arula1/Arula2	Herrera-Foessel et al. (2012)
Stem rust	*Sr2*	STS, CAPS	*T. turgidum*	Chinese Spring (Hope 3B)	Spielmeyer et al. (2003), Hayden et al. (2004), Mago et al. (2011)
	*Sr9a*	SSR	*T. aestivum*		Tsilo et al. (2007)
	*Sr22*	RFLP	*T. monococcum*	*T. boeoticum*	Paull et al. (1995)
	*Sr24*	STS	*Agropyron elongatum*		Mago et al. (2005)
	*Sr25*	STS	*Thinopyrum ponticum*		Liu et al. (2010)
	*Sr26*	STS	*Agropyron elongatum*,	*Datatine*	Mago et al. (2005)
	*Sr28*	PCR	*_*	Kota,Ceres and Line AD	Rouse et al. (2012)
	*Sr35*	SSR	*T. aestivum*	CRL-Sr35	Babiker et al. (2009); http://maswheat.ucdavis.edu/protocols/Sr35/index.htm
	*Sr38*	STS/CAPS	*A. Ventricosa*		Helguera et al. (2003)
	*Sr39*	STS	*A. speltoides*	Sr39#22r	http://maswheat.ucdavis.edu
	*Sr36*	SSR	*T. timopheevi*	Sr36/9^∗^LMPG	Tsilo et al. (2008)
	*Sr47*	SSR	*Aegilops speltoides*	RWG35, RWG36 and RWG37	http://maswheat.ucdavis.edu/protocols/Sr47/index.htm
	*Sr52*	STS	*D. villosum*		http://maswheat.ucdavis.edu/protocols/Sr52/
	*Sr R*	STS	*Secale cereale*		Mago et al. (2002)
	*Sr32*	SSR	*A. speltoides*	Chinese Spring	Mago et al. (2013)
	*Sr43*	SSR	*T. aestivum*	KS10-2, KS24-1	Niu et al. (2014)
	*Sr45*	SSR/AFLP	*T. aestivum*	CS1D5406	Periyannan et al. (2014)
	*Sr54*	SSR	*Ae. tauschii*		Yu et al. (2015)
	*Sr56*	STS and SSR	*T. aestivum*	Yitpi	Bansal et al. (2014)
Stripe rust	*Yr5*	STS	*T. spelta*		Chen et al. (2003), Yan et al. (2003)
	*Yr10*	SSR, STS	*T. aestivum*	P.I.178383	Wang et al. (2002), Singh et al. (2009)
	*Yr15*	SSR	*T. dicoccoides*		Peng et al. (2000)
	*Yr17*	STS/CAPS, SCAR	*A. Ventricosa*	RL 6081	Robert et al. (1999), Helguera et al. (2003)
	*Yr26*	SSR, EST-STS	*H. Villosa, Brachypodium distachyon*	R55, 92R137	Ma et al. (2001), Zhang et al. (2013)
	*Yr28*	RFLP	*T. aestivum*	Schmal/Opata 85’	Singh et al. (2000)
	*Yr50*	5 SSR	*T. aestivum*	CH223	Liu et al. (2013)
	*Yr51*	DArT(Marker sun104)	*T. aestivum*	AUS27858	Randhawa et al. (2013)
	*YrH52*	SSR	*T. dicoccoides*	Hermon H52	Peng et al. (2000)
	*Yr53*	RGAP/SSR	*T. aestivum*	PI 480148’	Xu et al. (2013)
	*Yr59*	RGAP and SSR	*T. aestivum*	PI 178759’	Zhou et al. (2014c)
	*Yr61*	STS5467 and STS5765b,	*T. aestivum*	Pindong 34.	Zhou et al. (2014a)
	*Yr64*	SSR	*T. aestivum, T. durum*	PI 331260 and PI 480016	Cheng et al. (2014)
	*Yr65*	SSR	*T. aestivum, T. durum*	PI 331260 and PI 480016	Cheng et al. (2014)
	*YrSD*	SSR	*T. aestivum*	Strubes Dickkopf	Jing et al. (2013)
	*YrHA*	SSR	*T. aestivum*	H9014-121-5-5-9	Ma et al. (2013)
	*YrSN104*	SSR	*T. aestivum*	Shaannong 104	Asad et al. (2012)

Cloning of resistance genes is an important approach for providing molecular insights and increasing resistance durability against rust resistance (Ellis et al., 2014; Jonathan et al., 2014). Lawrence et al. (1995) cloned first rust resistance gene *L6* from flax (linseed). In case of cereal, *Rp1-d* was the first rust resistance gene to be cloned by Collins et al. (1999) from corn. More than 30 resistance genes have been cloned in common wheat including *Lr10, Lr1, Lr21* for leaf rust (Huang et al., 2003; Cloutier et al., 2007; Loutre et al., 2009; Liu et al., 2012). The resistance genes are ineffective individually to the upcoming pathotypes of rusts in the world, thus pyramiding different resistance genes to breed multiline cultivars may increase the durability of resistance (Wen et al., 2008). Two highly effective genes for leaf rust resistance viz., *Lr24, Lr28* and a stripe rust resistance gene *Yr15* were selected for pyramiding in the susceptible but high yielding Indian bread wheat variety HD2877 (Revathi et al., 2010). Three highly effective leaf rust resistance genes, *Lr 24, Lr 28*, and *Lr 9* were selected for pyramiding in the bread wheat variety HD 2329 of India (Charpe et al., 2012). Vanzetti et al. (2011) reported that combinations of *Lr16, Lr47, Lr19, Lr41, Lr21, Lr25*, and *Lr29*, with *Lr34, SV2, Lr46* provide durable and effective resistance to leaf rust. An alternative and efficient strategy to detect quantitative trait loci (QTL) is association mapping (AM) or linkage disequilibrium (LD)-based mapping, in which genotype–phenotype relationships are explored in genetically diverse germplasm (Flint-Garcia et al., 2003; Zhu et al., 2008). AM has proved to be an efficient approach for both tetraploid and hexaploid wheat, by which enhancing previously available QTL information for MAS (Breseghello and Sorrells, 2006; Maccaferri et al., 2011). For leaf rust, QTLs were identified in 164 elite durum wheat accessions from different countries using AM approach (Maccaferri et al., 2010).

## Stem Rust

Stem or black rust is a major disease caused by fungus *P. graminis* f. sp. *tritici*. Wheat, durum wheat, barley, triticale, barley grasses (*Hordeum* sp.) and common wheat grass (*Agropyron scabrum*) are among the most commonly infected crops by stem rust. The Italians Fontana and Tozzetti independently provided the first report on stem rust in wheat in 1767. In large areas of the world, the life cycle of *P. graminis* consists of continual uredinial generations. The disease either spreads via airborne spores or occasionally from local-wild susceptible barberry (*Berberis* sp.) plants (Eversmeyer, 2000). Wheat (primary host) and barberry (secondary host) are required to complete the life cycle of fungus (Leonard and Szabo, 2005). Five types of spores (pycniospores, aeciospores, urediniospores, teliospores, and basidiospores) occur in the life cycle of fungus at different developmental stages (Leonard, 2001). Warm temperature (15–30°C) and dew are the two important factors favoring the crop infection by stem rust. Stem rust usually occurs on the stem, and can also occur on the leaves (both sides), leaf sheaths or in severe infections on the head. Uredia pustules on stem and leaf sheaths are the main symptoms of disease spreading (Leonard, 2001). Reddish brown color and oval or spindle-shaped pustules are seen on the stem and leaf sheath. Pustules would change to black in color at the end of the season when infection is too old (Todorovska et al., 2009) and can cause severe crop loss in a short span of time at the end of the season.

In the early to mid 1950s; stem rust epidemics caused approximately 50% yield losses of wheat in North America (Leonard, 2001). During 1950s, Norman Borlaug and other scientists started developing high-yielding wheat varieties that were resistant to stem rust and other diseases in North America and throughout the world (Singh et al., 2006). Resistant plants exhibit no or less number of uredia surrounded by chlorosis or necrosis as compared to susceptible plants. A new race of stem rust (Ug99) causing a high level of infection on wheat genotypes was found in 1999 in Uganda (Pretorius et al., 2000). Heavy stem rust infections were observed in International Center for Wheat and Maize Improvement (CIMMYT)-derived lines of wheat in Kenya in 2004 (Kolmer, 2005; Todorovska et al., 2009). This race has spread to major wheat growing regions of the world such as Iran, Afghanistan, India, Pakistan, Turkmenistan, Uzbekista, Kazakhstan, USA, and Canada (Todorovska et al., 2009). Therefore it is necessary to develop a resistant germplasm to overcome the spreading of infection in these regions.

Since, breeding program in wheat for developing stem rust resistance is a challenging task for a breeder; therefore, acquisition of genetic resistance is the best alternative for controlling rust epidemics. Currently, about fifty stem rust resistance (*Sr*) genes have been identified. Moreover, mapping of few genes and their close relatives on different chromosomes of wheat has also been achieved (McIntosh et al., 1998). PCR (STS) and non-hybridization based (RFLP) markers are available for screening the genotypes which are resistant to stem rust disease (William et al., 2008). The molecular markers associated with *Sr* genes known so far are summarized in (**Table 1**). RFLP (*Sr22*-Paull et al., 1995) and STS (*Sr2*-Hayden et al., 2004; *Sr24, Sr26*- Mago et al., 2005; *Sr*R-Mago et al., 2002; *Sr*39-Mas wheat ucdavis), STS/SSR (*Sr56*-Bansal et al., 2014), SSR/AFLP (*Sr45*- Periyannan et al., 2014) STS/CAPS (*Sr38*-Helguera et al., 2003) and SSR (*Sr32*- Mago et al., 2013; *Sr43*-Niu et al., 2014; *Sr54*- Yu et al., 2015) markers have been reported to be associated with different *Sr* genes in wheat. *Sr2* is one of the non-race specific genes which have resulted in successful acquisition of durable rust resistance to slow rusting adult (Singh et al., 2004). It has been widely used by CIMMYT, Mexico in its wheat program for improvement of stem rust resistance and also in USA for hard winter wheat breeding program. Above all, the *Sr2* complex when used in combination with other resistance genes has shown remarkable protection against *Ug99* (Singh, 1993). CIMMYT and International center for agricultural research in the dry areas (ICARDA) started the global rust initiative (Later in 2008, BGRI- Borlaug global rust initiative) to coordinate efforts to track and study *Ug99* and develop resistant varieties of wheat (Stokstad, 2007). Some genes like *Sr33* and *Sr35* for stem rust resistance were cloned with the objective to increase resistance (Periyannan et al., 2013; Saintenac et al., 2013) Various studies have been conducted to confirm the presence of *Sr* genes in wheat cultivars. A recombinant inbred line (RIL) population of 83 lines (developed from a cross from Indian wheat cultivars VL404 and WL711) was screened to identify *Sr28* gene using SSR markers (Bansal et al., 2012). Haile et al. (2013) screened 58 tetraploid wheat accessions of Ethiopian wheat cultivars for the presence of 30 *Sr* genes using SSR and STS markers. 88 spring soft wheat of Kazakhstan were studied for presence of *Sr* genes (*Sr2, Sr22, Sr24, Sr36*, and *Sr46*) which are effective against Ug99 (Kokhmetova and Atishova, 2012). Thirty-seven lines of American cultivars with known stem rust resistance genes and five genetic background cultivars were used to further validate the six co-dominant STS markers for *Sr25* and *Sr26* (Liu et al., 2010). Mago et al. (2011) used DNA markers to check the presence of *Sr24, Sr26, SrR*, and *Sr31* in wheat-rye recombinant T6-1. These *Sr* genes provide resistance against all strains of stem rust that are prevalent in Australia. However, *Sr26* and *SrR* are effective outside Australia against strain Ug99. 104 F_2:3_ population of Gabo 56 with susceptible cultivar Chinese Spring were screened to check the presence of *Sr9h* using SSR markers. Minor stem rust resistance gene *Sr2* was pyramided with two major stem rust resistance genes *Sr24* and *Sr36* in Indian wheat varieties ‘Lok-1’ and ‘Sonalika’ (Nisha et al., 2015). AM study for response to stem rust was conducted on 183 Ethiopian durum wheat accessions and 276 wheat lines from Kenya (Yu et al., 2011; Letta et al., 2013).

## Yellow Rust or Stripe Rust

Stripe or yellow rust, caused by *P. striiformis* f. sp. *tritici*, mainly infects wheat, but can also cause infection in barley, rye, and triticale. It was first reported in USA (Carleton, 1915) and outbreaks were reported in the Western states in 1960s (Boyd, 2005). Later on, the infections were also reported from other parts of the of world including USA, East Asia (China north-west and southwest), South Asia (India, Pakistan, and Nepal), Oceania (Australia, New Zealand), East Africa (Ethiopia, Kenya), the Arabian Peninsula (Yemen) and Western Europe (Wellings, 2011). Presently, more than 35% of area under wheat cultivation is affected by stripe rust disease (Singh et al., 2004). Cool and wet weather is favorable for the development of yellow rust. Pustules are light yellow and occur on leaves in distinct straight-sided stripes about 1/16 inches wide and of regular length. The spores are yellow to orange in color. Reduced dry matter production, root growth, plant height, size and number of flowering spikes, and the size and number of grains are the parameters affected by infection. These effects were more pronounced with infection beginning at the seedling stage, although infections initiated at anthesis were also associated with reduced root weight and grain yield (Wellings, 2011).

Breeding efforts for stripe rust resistance has been made in the past. Breeding approaches involves developing several crosses with careful phenotypic selection which makes it difficult for a breeder to achieve the desired objective. About 52 permanently named and more than 40 temporarily designated genes or QTL for stripe rust resistance have been reported (Chen, 2005; McIntosh et al., 2011; Ren et al., 2012). Among the permanently named resistance genes, *Yr11, Yr12, Yr13, Yr14, Yr16, Yr18, Yr29, Yr30, Yr34, Yr36, Yr39, Yr46, Yr48*, and *Yr52*, confer adult plant or high temperature adult plant (HTAP) resistance genes, whereas the others confer all-stage resistance. The identification and use of the resistant genes is the only way to conquer the impact of disease on wheat production. Till date, 65 (*Yr1*–*Yr65*) yellow rust resistance genes have been characterized and designated in wheat (McIntosh et al., 1995; Singh et al., 2004; Boyd, 2005; McIntosh et al., 2008). A wide range of markers are reported to be associated with *Yr* genes (**Table 1**). RFLP (*Yr28*-Singh et al., 2000), SSR (*Yr10*-Wang et al., 2002; *Yr15, Yr26, YrH52*-Peng et al., 2000), STS/CAPS (*Y17*-Robert et al., 1999; Helguera et al., 2003; *YrMoro*-Smith et al., 2002), STS (*Yr61*-Zhou et al., 2014a), DArt (*Yr51*-Randhawa et al., 2014), RGAP/SSR (*Yr59*-Zhou et al., 2014c) and SSR (*YrSN104*-Asad et al., 2012; *Yr 50*- Liu et al., 2013; *Yr64* and *Yr65*-Cheng et al., 2014) markers have been reported to be associated with different *Yr* genes in wheat. Most of the identified yellow rust resistant *Yr* genes have been characterized as the race specific ones and are responsible for acquiring resistance against the isolates of *P. striiformis* f. sp. *tritici* only, which carries the corresponding avirulence (*avr*) gene. Various stripe rust resistant genes have been transferred into hexaploid wheat from different wild species (Kuraparthy et al., 2007a,b; Singh et al., 2007; Chhuneja et al., 2008). With the help of molecular marker a study reveals that recent Canadian wheat varieties have the strip rust resistant genes *Yr* 10, *Yr*17, *Yr*18, and *Yr* 36 (Randhawa et al., 2012). Further, a highly stripe rust resistant gene, namely *Yr36* has been used for positional cloning. *Yr36* gene, derived from wild emmer wheat, carries broad spectrum resistance for stripe rust races (Fu et al., 2009). A total of 54 wheat genotypes representing breeding lines and current grown cultivars in the western US were tested with race PST-100 and the *Yr53*-flanking markers, XLRRrev/NLRRrev350, Xgwm441 and the STS marker (STS2F/1R219) developed from RGAP marker, Ptokin2/Xa1NBSF234 (Xu et al., 2013).

Four Gatersleben wheat microsatellite (GWM) markers were used to identify non-specific adult plant disease resistance genes against stripe rust in 160 F_2_ plants from the cross of UK/German wheat cultivars Lgst.7/Winzi (Khlestkina et al., 2007). To identify genes for stripe rust in 181 plants from one segregating F_3_ line of Xiaoyan/Mingxian cross. SSR primers were used to identify molecular markers flanking *Yrxy2*, whereas for *Yrxy1* RGAP and SSR markers both were used (Zhou et al., 2011). Naz et al. (2012) done QTL analysis by using a genetic map based on 118 SSR markers in 150 back cross lines of German wheat cultivars Zentos and Syn86L. To identify genes for stripe rust resistance in 179 F_2_ population of Wuhan 2/Mingxian 169 cross against races CYR30 and CYR31 using RGAP and SSR markers (Zhou et al., 2014b). Yaniv et al. (2015) concluded from their findings that SSR markers from *Yr15* region are efficient tools for MAS and for introgression of *Yr15* into wheat from *T. dicoccoides*. In case of stripe rust resistance genes, *Yr17, Yr18*, and *Yr36* were amongst the successfully cloned genes (Helguera et al., 2003; Lagudah et al., 2009; Fu et al., 2009). Stripe rust response for adult plants was evaluated using AM in 192 genotypes including 181 synthetic hexaploid wheat (SHW) and 11 bread wheat cultivars from different countries (Zegeye et al., 2014). Similar studies were performed using 402 wheat varieties and 1000 spring wheat accessions from USA (Naruoka et al., 2015; Maccaferri et al., 2015).

## Recent Trends

Recently the new technologies are being used for sequencing of cereal crops, but the storage of data and analysis are difficult due to its vast size. Single nucleotide polymorphism (SNP) genotyping offers a solution to this problem and accelerates the crop improvement by providing insights into their genetic constitution. It has number of advantages over conventional marker system such as rapid processing of large populations, abundance of markers and varieties of genotyping system (Thomson, 2014). In quantitative trait locus (QTL) mapping experiments and genome-wide association studies (GWAS), SNP data is frequently used to detect marker-trait associations (Zhao et al., 2011; Cook et al., 2012). Discovery of SNPs using complete genome is facilitated by recent advances in next-generation sequencing (Berkman et al., 2012; Chia et al., 2012; Xu et al., 2012). Genetic studies of number of economically important crops have been successfully done by the application of high-density SNP arrays (Wiedmann et al., 2008; Ganal et al., 2011; Zhao et al., 2011; Sim et al., 2012; Song et al., 2013). 44K SNP genotyping chip was employed for GWAS of diverse rice accessions and identified number of alleles responsible for governing morphological and agronomic traits (Zhao et al., 2011). Similarly, the genetic control of maize kernel composition in a nested AM panel was studied by the use of 50K maize SNP chip (Cook et al., 2012; Hufford et al., 2012). Moreover, the genomic regions targeted by breeding in wheat were detected by 9K SNP wheat (Cavanagh et al., 2013). The most challenging task is to analyze the genotypic data of durum [*T. turgidum* subsp. durum (Desf.) Husnot] and bread wheat (*T. aestivum* L.) genome using SNP genotyping platforms (Akhunov et al., 2009). The use of wheat SNP iSelect array has proven to be a promising tool to infer detailed haplotype structure in polyploid wheat and will serve as an invaluable resource for diversity studies and investigating the genetic basis of trait variation in wheat. A combination of eight mapping populations was used to genetically map 46,977 SNPs using wheat 90K array (Wang et al., 2014).

## Conclusion

Due to global food security and consistent increase in world population, there is an immediate need to increase wheat yield considerably. Fungal diseases continue to cause huge losses and pose a great challenge for wheat production. Novel genetic tools based on molecular marker technologies provide a good alternative for developing improved resistant cultivars. Development of molecular markers such as RFLPs, SSRs, AFLPs, SNPs, and DArT in last more than two decades has revolutionized wheat genomics. Marker assisted breeding and functional genomics tools are effective strategies to develop resistant cultivars against fungal diseases in wheat for achieving estimated production paradigm. In future, functional genomics approaches such as TILLING, RNAi and epigentics etc. are needed to strengthen the development of resistant varieties. Mutagenesis-derived broad-spectrum disease resistance may lead to a better understanding of the regulation of defense response networks in wheat. Large-scale genome sequencing and associated bioinformatics are becoming widely accepted research tools for accelerating the analysis of wheat genome structure and function. Currently, functional markers are being increasingly adopted in wheat breeding. These markers are needed for important traits such as disease and stress resistance in order to strengthen the application of molecular markers in breeding programs. The collaborative effort (MASwheat: http://maswheat.ucdavis.edu/index.htm) by United States Department of Agriculture (USDA), National Institute of Food and Agriculture (NIFA) and Borlaug Global Rust Initiative (BGRI) has given the platform for transferring new developments in wheat genomics and biotechnology to increase wheat production. Many traits such as the disease/pest resistance and end-use quality which has increased the competitiveness of wheat breeding programs through MAS were included. Triticeae Coordinated Agricultural Project (T-CAP) focused on studying the effects of climate change on crop yields by identification and incorporation of genetic loci for enhancing tolerance in crops. For improving the barley and wheat germplasm, gene variants for disease resistance, water and nitrogen use efficiency and yield improvement are being identified, along with molecular markers to tag them and accelerate breeding. The International Wheat Genome Sequencing Consortium (IWGSC) will put the foundation to accelerate wheat improvement for wheat growers, scientists, and breeders. The ultimate goal leads to obtain high quality annotation of the genome and thus complete sequencing of the common wheat genome.

## Conflict of Interest Statement

The authors declare that the research was conducted in the absence of any commercial or financial relationships that could be construed as a potential conflict of interest.
